# Endogenous Uteroglobin as Intrinsic Anti-inflammatory Signal Modulates Monocyte and Macrophage Subsets Distribution Upon Sepsis Induced Lung Injury

**DOI:** 10.3389/fimmu.2019.02276

**Published:** 2019-10-01

**Authors:** Andrea Janicova, Nils Becker, Baolin Xu, Sebastian Wutzler, Jan Tilmann Vollrath, Frank Hildebrand, Sabrina Ehnert, Ingo Marzi, Philipp Störmann, Borna Relja

**Affiliations:** ^1^Department of Trauma, Hand and Reconstructive Surgery, Goethe University, Frankfurt, Germany; ^2^Department of Aquatic Ecotoxicology, Goethe University, Frankfurt, Germany; ^3^Department of Radiology and Nuclear Medicine, Experimental Radiology, Otto-von-Guericke University, Magdeburg, Germany; ^4^Orthopedic and Trauma Surgery, Helios Horst Schmidt Clinic, Wiesbaden, Germany; ^5^Department of Trauma Surgery, RWTH University, Aachen, Germany; ^6^Department of Trauma and Reconstructive Surgery, Siegfried Weller Research Institute, BG Trauma Center Tuebingen, Eberhard Karls University Tuebingen, Tuebingen, Germany

**Keywords:** uteroglobin, CC16, chest injury, acute lung injury, CLP, sepsis, monocytes, macrophages

## Abstract

Sepsis is a serious clinical condition which can cause life-threatening organ dysfunction, and has limited therapeutic options. The paradigm of limiting excessive inflammation and promoting anti-inflammatory responses is a simplified concept. Yet, the absence of intrinsic anti-inflammatory signaling at the early stage of an infection can lead to an exaggerated activation of immune cells, including monocytes and macrophages. There is emerging evidence that endogenous molecules control those mechanisms. Here we aimed to identify and describe the dynamic changes in monocyte and macrophage subsets and lung damage in CL57BL/6N mice undergoing blunt chest trauma with subsequent cecal ligation and puncture. We showed that early an increase in systemic and activated Ly6C^+^CD11b^+^CD45^+^Ly6G^−^ monocytes was paralleled by their increased emigration into lungs. The ratio of pro-inflammatory Ly6C^high^CD11b^+^CD45^+^Ly6G^−^ to patrolling Ly6C^low^CD11b^+^CD45^+^Ly6G^−^ monocytes significantly increased in blood, lungs and bronchoalveolar lavage fluid (BALF) suggesting an early transition to inflammatory phenotypes during early sepsis development. Similar to monocytes, the level of pro-inflammatory Ly6C^high^CD45^+^F4/80^+^ macrophages increased in lungs and BALF, while tissue repairing Ly6C^low^CD45^+^F4/80^+^ macrophages declined in BALF. Levels of inflammatory mediators TNF-α and MCP-1 in blood and RAGE in lungs and BALF were elevated, and besides their boosting of inflammation via the recruitment of cells, they may promote monocyte and macrophage polarization, respectively, toward the pro-inflammatory phenotype. Neutralization of uteroglobin increased pro-inflammatory cytokine levels, activation of inflammatory phenotypes and their recruitment to lungs; concurrent with increased pulmonary damage in septic mice. In *in vitro* experiments, the influence of uteroglobin on monocyte functions including migratory behavior, TGF-β1 expression, cytotoxicity and viability were proven. These results highlight an important role of endogenous uteroglobin as intrinsic anti-inflammatory signal upon sepsis-induced early lung injury, which modules the early monocyte/macrophages driven inflammation.

**Short Summary:**

Blunt chest injury is the third largest cause of death following major trauma, and ongoing excessive pro-inflammatory immune response entails high risk for the development of secondary complications, such as sepsis, with limited therapeutic options. In murine double hit trauma consisting of thoracic trauma and subsequent cecal ligation and puncture, we investigated the cytokine profile, pulmonary epithelial integrity and phenotypic shift of patrolling Ly6C^low^CD11b^+^CD45^+^Ly6G^−^ monocytes and Ly6C^low^CD45^+^F4/80^+^ macrophages to pro-inflammatory Ly6C^high^CD11b^+^CD45^+^Ly6G^−^ monocytes and Ly6C^high^CD45^+^F4/80^+^ cells in blood, lungs and bronchoalveolar lavage fluid (BALF). Pro-inflammatory mediators and phenotypes were elevated and uteroglobin neutralization led to further increase. Enhanced total protein levels in BALF suggests leakage of respiratory epithelium. *In vitro*, uteroglobin inhibited the migratory capacity of monocytes and the TGF-β1 expression without affecting the viability. These results highlight an important role of endogenous uteroglobin as an intrinsic anti-inflammatory signal upon sepsis-induced early lung injury, which modulates the early monocyte/macrophages driven inflammation.

## Introduction

Trauma is one of leading causes of death worldwide (1). Twenty to twenty-five percent of patients with multiple injuries suffer from severe lung contusion, whereas thoracic trauma represents the third most frequent cause of death after major trauma (2, 3). Thoracic injury contributes significantly to the development of acute respiratory distress syndrome (ARDS) (4), while infectious complications such as e.g., sepsis constitute a serious risk factors for up to 50% of mortalities occurring upon ARDS (5). Thus, the development of secondary complications is still a major contributing factor in trauma-associated mortality (6–8).

In general, traumatic injury-related tissue damage induces a release of endogenous damage-associated molecular patterns to initiate the resolution of non-pathogenic and pathogenic inflammation with subsequent tissue repair (9). This systemic inflammatory response syndrome triggers an excessive release of pro-inflammatory cytokines such as tumor necrosis factor (TNF)-α (9), interleukin (IL)-6 (9) and chemokines, such as monocyte chemoattractant protein (MCP)-1 (10, 11), which is a potent factor for monocyte and macrophage migration and infiltration (12).

Monocytes play a pivotal role in pathogen recognition and killing, and although their functional and phenotypic alterations provide a good base for prediction of complications after trauma or of poor prognosis in septic patients, exact pathophysiologic mechanisms and solid biomarkers still remain to be elucidated (13–17). Murine monocytes expressing high levels of lymphocyte antigen (Ly)6C have pro-inflammatory and anti-microbial features and have been shown to be precursors for patrolling monocytes, which survey the vasculature and contribute to the early response of inflammation and tissue repair, and which are characterized by a low expression of Ly6C (18, 19). Next to monocytes, resident alveolar macrophages initiate the inflammatory cascade and secrete pro-inflammatory mediators during acute lung injury (ALI) (20–22). While the pro-inflammatory M1 macrophages release e.g., nitric oxide, TNF-α, interferon-γ and IL-12 and critically contribute to pathogen clearance, their apoptosis during the process of pathogen clearance simultaneously contributes to downregulation of the pro-inflammatory phase and transition of M1 to tissue-repairing M2 macrophages (23). With regards to specific roles of monocytes/macrophages during lung injury and/or sepsis, both beneficial or detrimental effects of each cell type have been reported. While an early depletion of circulating monocytes before lipopolysaccharide (LPS) administration deteriorated lung injury (24), later monocyte depletion ameliorated lung injury (25, 26). Furthermore, macrophage polarization into M1 phenotype improved organ dysfunction and reduced mortality in lethal sepsis (27), while an intratracheal administration of M2 macrophages after CLP reduced mortality (28). These results indicate that an early balance of the pro-inflammatory and the anti-inflammatory response is required for ameliorating lung injury.

Uteroglobin (club cell protein (CC)16), is a 15.8 kDa protein secreted primarily by non-ciliated club cells along the tracheobronchial epithelium, especially in distal respiratory and terminal bronchioles (29, 30). Next to its biomarker character to indicate the development of secondary pulmonary complications after trauma, CC16 exerts anti-inflammatory and immunosuppressive properties (31–33). Its anti-inflammatory biology has been confirmed in tracheal epithelial cells, isolated human mononuclear cells and murine macrophages (34–36). Due to this, CC16 has been described as being protective in the development of chronic obstructive pulmonary disease in human (37) and mouse (33).

Considering that functional and phenotypic alterations of monocytes/macrophages play an important role in sepsis development and due to the potent anti-inflammatory biology of CC16, we hypothesize that an early upregulation of the pro-inflammatory response by local CC16 neutralization will deteriorate the dynamic changes in monocyte and macrophage subsets and early lung damage in a murine trauma model of sepsis after blunt chest trauma.

## Materials and Methods

### Ethics

The *in vitro* study was performed in the University Hospital Frankfurt, Goethe-University, Germany, with the institutional ethical committee approval (312/10) in accordance with the Declaration of Helsinki and following STROBE-guidelines (38). In this experimental trial, twenty severely injured trauma patients (TP) with a history of acute blunt or penetrating trauma with an injury severity score (ISS) of ≥ 16 were enrolled, along with and 8 healthy volunteers. All individuals who were <18 or >80 years of age, suffering from a severe burn injury, acute myocardial infarction, cancer or chemotherapy, HIV, infectious hepatitis, acute CMV infection and/or thromboembolic events, or receiving immunosuppressive drug therapy were excluded. The ISS was calculated according to the abbreviated injury scale (39) upon arrival to the emergency department. The signed written informed consent form was obtained from all patients or their legally authorized representatives, as well as from all included healthy volunteers (HV).

Animal experiments were conducted at the Zentrale Forschungseinrichtung of the University Hospital Frankfurt in accordance with the German Federal Law in regard of protection of animals with the approval of the responsible government authority, the Veterinary Department of the Regional Council in Darmstadt, Germany (Regierungspräsidium Darmstadt, Hessen, Germany; AZ: FK 1068). All experiments were performed in accordance with the ARRIVE Guidelines (40).

### Animals and Experimental Model

Forty male CL57BL/6N mice (25 ± 5 g, 6–8 weeks old) were included (Janvier Labs, France) (41). Before and after experimental procedures, all animals had access to water and food *ad libidum*. Blunt chest trauma was performed under general mask anesthesia as described before (41). Briefly, the animals were placed in a supine position and a blunt bilateral thoracic trauma (TxT) was induced by a standardized pressure wave provided directly to the chest (41). After 24 h, a median laparotomy with moderate cecal ligation and puncture (CLP) followed as described before (41). Eight animals underwent only TxT. Twenty-four animals underwent the double hit consisting of TxT and CLP. Eight animals in the sham control group underwent anesthesia without performing any surgical procedures. After 6 h, euthanasia was done to facilitate sampling.

### Group Allocation Based on the Administration of CC16 Neutralizing Antibody

Animals were randomly assigned to different experimental groups for local antibody (Ab) application to the lungs. Administration of Uteroglobin/SCGB1A1 (CC16 Ab, 10 μg/mL, LS Biosciences) or IgG Control (IgG) Antibody (10 μg/mL, R&D Systems) was performed immediately after the induction of thoracic trauma. For this procedure, mice were placed in a supine position and the tongue was thoroughly kept aside. A buttoned cannula was placed at the beginning of the trachea and 50 μL of the Ab solution were carefully administered. Then, mice were kept in a reverse trendelenburg position for 30 s to ensure the Ab distribution inside the lungs.

### Sampling and Quantification of Protein Expression Levels in Lungs, Plasma, and BALF

The *vena cava* was punctured by a heparinized syringe for blood withdrawal at 6 h after CLP. After centrifugation at 1,164 g for 15 min at 4°C, the plasma was stored at –80°C for the subsequent measurements of pro-inflammatory mediators. MCP-1 and TNF-α were measured in plasma with the CBA Mouse Inflammation Kit (BD Bioscience, San Jose, CA, USA) according to the manufacturer's instructions. Briefly, 50 μL of the Capture Beads were added into polystyrene FACS tubes (BD Pharmingen™) to 50 μL of plasma. To each FACS tube, 50 μL of the Mouse Inflammation PE Detection Reagent were added and incubated at room temperature in the dark for 2 h. Subsequently, samples were washed with 1 mL of Wash Buffer and centrifuged at 200 g for 5 min. Supernatant was discarded and pellet resuspended in 300 μL of Wash buffer. Analysis was performed using a BD FACS Canto 2™ and FCAP Array™Software (BD).

After blood withdrawal, the trachea was punctured, intubated and the lungs were flushed with 1.2 mL phosphate buffered saline (PBS) to gain the bronchoalveolar lavage fluid (BALF) for analysis. BALF samples were centrifuged at 1,164 g at 4°C for 5 min and the supernatant was used for the detection of the receptor for advanced glycation endproducts (RAGE DuoSet® ELISA Kit; R&D Systems, Minneapollis, US). Quickly, a microplate (Sarstedt, Nümbrecht, Germany) was coated with 100 μL Capture Antibody overnight at room temperature. Following a washing step, 300 μL of Reagent Diluent was added for 1 h to block the microplate. After another washing step, samples were loaded and incubated for 2 h at room temperature. Subsequently, the microplate was washed again and incubated with 100 μL Detection Antibody for 2 h. Microplate was washed again and incubated with 100 μL Streptavidin-HRP solution for 20 min in the dark at room temperature. Following the last washing step, 100 μL Substrate Solution was added to the wells and incubated in the dark at room temperature until a color reaction occurred. Subsequently, the reaction was stopped by adding 50 μL Stop Solution. The optical density was measured with the Infinite M200 microplate reader (Tecan, Männedorf, Switzerland, 450 nm absorbance, 570 nm reference wavelength; software Magellan).

The cell pellets from BALF were resuspended in 100 μL PBS supplemented with 0.5% bovine serum albumin (FACS buffer), and 40 μL were transferred into polystyrene FACS tubes (BD Pharmingen™) for subsequent cell staining as described below.

Then the animals were perfused with 20 mL PBS via the caudal *vena cava*, and, subsequently, the lungs were removed. One lung lobe was snap-frozen using liquid nitrogen for later protein isolation, and the other one was used for flow cytometric analyses. For protein isolation, lung tissue was homogenized in protein lysis buffer at 4°C, followed by centrifugation for 30 min at 4°C at 20,000 g. Supernatants were stored at –80°C for later analysis. Protein concentrations of pulmonary RAGE were determined using a mouse RAGE DuoSet® ELISA Kit (R&D Systems) as described above.

### Analysis of Monocyte and Macrophage Subsets by Flow Cytometry

Lung tissue was processed as described in the Minute Single Cell Isolation protocol (Invent Biotechnologies, Minnesota, US). Briefly, 25 mg of fresh lung tissue were placed into a filter cartridge where 100 μL ice-cold Buffer A were subsequently added. The tissue was grinded with a plastic rod for 50–60 times. After adding further 400 μL Buffer A, sample was mixed by inverting the closed filter cartridge and centrifuged at 1,200 g and 4°C for 5 min. The pellet was resuspended and centrifuged again at 400 g and 4°C for 5 min. Subsequently, supernatant was discarded and pellet was resuspended in 100 μL FACS buffer. Forty microliter were transferred into each polystyrene FACS tubes (BD Pharmingen™) and stained for flow cytometry analysis as described below.

Thirty microliters of whole blood was transferred into each polystyrene FACS tubes (BD Pharmingen™) and stained for flow cytometry analysis as described below.

The cell pellets from BALF were resuspended in 100 μL of FACS buffer, and 40 μL was transferred into polystyrene FACS tubes (BD Pharmingen™).

Then, the samples were incubated with Pacific Blue-conjugated anti-mouse Ly-6G/Ly6C antibody (Ab) (Clone RB6-8C5; BioLegend, San Diego, California, US), APC/Fire 750 conjugated anti-mouse CD45 Ab (Clone 30-F11; BioLegend), Alexa Fluor 647-conjugated anti-mouse CD11b Ab (Clone M1/70; BioLegend), Brilliant Violett 510-conjugated anti-mouse F4/80 Ab (Clone BM8; BioLegend), and Phycoerythrin-Cyanine7-conjugated anti-mouse Ly6C Ab (Clone RB6-8C5; BioLegend). Control stainings with the corresponding isotype antibodies were applied for the settings. After 30 min on ice, 5 μL of 7-AAD (BD Biosciences, Franklin Lakes, USA) were added, and samples were incubated for further 15 min. Then, the samples were washed with 2 mL FACS buffer [7 min at room temperature (RT) and 423 g]. Supernatants were removed and cell pellets were homogenized in 1 mL of BD FACS Lysing Solution for an additional 10 min (RT). Then, samples were centrifuged at 400 g for 7 min and washed twice with 2 mL of FACS buffer. After removal of supernatants, cells were diluted in 80 μL FACS buffer and stored on ice until measurement. Each cell population was defined by gating the corresponding forward and side scatter scan as well as the viable cells by applying 7-AAD for gating. From each sample a minimum of 3.0 x 10^4^ cells was measured, which were subsequently analyzed. The percentage of Ly6C^+^ out of CD11b^+^Ly6G^−^CD45^+^ and F4/80^+^CD45^+^ viable cells was assessed by flow cytometric analyses using a BD FACS Canto 2™ and FACS DIVA™ software (BD). The gating is shown in **Figure 2**.

### Quantification of Uteroglobin in Sera From Healthy Volunteers and Trauma Patients

Collected sera from healthy volunteers and trauma patients were analyzed using human Uteroglobin Quantikine ELISA Kit (R&D Systems, Minneapolis, US) according to the manufacturer's instructions. Briefly, 100 μL of Assay Diluent were added to each well with subsequent addition of 50 μL of each sample and incubated at room temperature for 2 h. Then, each well was washed with 400 μL Wash Buffer. Subsequently, wells were incubated with 200 μl of Human Uteroglobin Conjugate for 2 h. After the next washing step, 200 μL of Substrate Solution were added into the wells for 30 min. The reaction was stopped by addition of 50 μl of Stop Solution to each well. The optical density was measured with the Infinite M200 microplate reader (Tecan, Männedorf, Switzerland, 450 nm absorbance, 570 nm reference wavelength; software Magellan).

### Isolation of CD14^+^ Monocytes

Isolation of peripheral blood mononuclear cells was performed by a density-gradient centrifugation (Bicoll separating solution, Biochrom, Berlin, Germany) according to manufacturer's instructions. Briefly, 25 mL of Bicoll separating solution (density: 1.077 g/mL) was carefully overlaid with an equal volume of heparinized whole blood from HV and centrifuged at 800 g for 30 min. Interphase containing peripheral blood mononuclear cells was transferred to another tube and washed with PBS w/o Ca^2+^ and Mg^2+^ (Invitrogen, Carlsbad, California, US). The remaining red blood cells were lysed by lysis buffer (0.155 M NH_4_Cl, 0.01 M KHCO_3_, 0.1 mM EDTA) and washed with MACS buffer (0.5% BSA, 2 mM EDTA). For CD14 labeling, cell pellet was resuspended in 75 μL of MACS buffer and incubated with 25 μL magnetic CD14 microbeads (Miltenyi Biotec, Bergisch Gladbach, Germany) for 15 min. After washing, CD14^+^ monocytes were isolated by magnetic isolation with LS columns (Miltenyi Biotec) according to the manufacturer's protocol. Cell number and cell viability were determined by Türk's solution exclusion assay (Merck, Darmstadt, Germany). Only cell cultures with a purity of ≥95% were used for further experiments. The cells were cultured in RPMI 1640 medium (Seromed, Berlin, Germany), supplemented with 10% heat-inactivated fetal calf serum (Gibco, Karlsruhe, Germany), 100 IU/mL penicillin (Gibco), 10 μg/mL streptomycin (Gibco) and 20 mM HEPES buffer (Sigma) at 37°C and 5% CO_2_.

### Monocyte Treatment

*Ex vivo*, CD14^+^ monocytes isolated from HV were treated with sera from HV and TP that were obtained at the admission to the emergency department. Prior to the experiment, the sera were incubated with or without anti-CC16-antibody (1 μg/mL; R&D Systems) for CC16 neutralization or corresponding isotype control antibody (1 μg/mL; R&D Systems), respectively, for 1 h at 37°C and 5% CO_2_, slightly slewing every 15 min. For monocyte stimulation, cell culture media was supplemented with 20% sera for 2 h at 37°C and 5% CO_2_ and used for further analysis.

### Migration Assay

Alterations in migratory capacity were determined by CytoSelect™ Cell Migration Assay (3 μm pores; Cell Biolabs, San Diego, US). 100,000 cells were plated in the upper chamber and treated as described in the Monocyte treatment section. MCP-1 (10 ng/mL; R&D Systems) was added to the lower chamber. After 3 h at 37°C and 5% CO_2_, the upper chamber was removed and cells in the lower chamber were lysed and quantified using CyQuant® GR Fluorescent Dye (Cell Biolabs, San Diego, US) according to the manufacturer's instructions. Briefly, the cells containing supernatant from the feeder tray was transferred into a black-walled, clear bottom microplate. CyQuant® GR Dye was diluted 1:75 in 4x Lysis Buffer and subsequently added to each well to reach a 1x concentration. Samples were incubated at RT for 20 min. Fluorescence intensity was measured by Twinkle LB 970 Microplate Fluorometer (490 nm excitation/520 nm emission; software MikroWin 2000).

### Measurement of TGF-β1 Expression in Monocytes by Flow Cytometry

100,000 cells per polystyrene FACS tube (BD Pharmingen™) were treated according to the Monocyte treatment section with slight change. After 1 h of treatment with sera, Brefeldin A (Invitrogen) was added to each tube to 1x concentration and monocytes were incubated for further 2 h at 37°C and 5% CO_2_. Subsequently, monocytes were incubated with Phycoerythrin-conjugated anti-human CD14 antibody (2 μL; Clone M5E2; BioLegend) and fixable yellow dead cell stain (2 μL; Invitrogen, Carlsbad, California, US). After 30 min at RT, cells were washed with FACS buffer and centrifuged at 400 g for 5 min. Supernatant was removed and monocytes were fixed with Fix and Perm Medium A at room temperature for 15 min. After further washing step, cells were permeabilized with Fix and Perm Medium B (both Invitrogen, Carlsbad, California, US) and incubated with PerCP/Cyanine5.5-conjugated anti-human TGF-β1 antibody (2 μL; Clone TW4-2F8; BioLegend) at room temperature for 30 min. Following the washing step, monocytes were resuspended in 50 μL FACS buffer and analyzed using BD FACS Canto 2™ and FACD DIVA™ software (BD). Monocytes were gated by the corresponding forward and side scatter scan and as shown in **Figures 5A,B**. The percentage of TGF-β1 expression of viable CD14^+^ monocytes was analyzed.

### Quantification of IL-6 and TNF-α Levels in Monocyte Supernatants

100,000 cells were seeded in flat-bottom 96-well plate (Sarstedt) and treated as described in the Monocyte treatment section. The supernatants were collected to detect the IL-6 (Diaclone, Besançon cedex, France) or TNF-α (R&D Systems, Minneapollis, US) levels using ELISA kits according to the provider's instructions. For a brief IL-6 protocol, see equivalent RAGE measurement protocol in the Sampling and quantification of protein expression levels in lungs, plasma and BALF section. The protocol for TNF-α is equivalent to the Uteroglobin Quantikine ELISA Kit described in the section Quantification of uteroglobin in sera from healthy volunteers and trauma patients.

### Cell Viability Assay

100,000 cells per well were plated in a clear bottom, black-walled 96-well plate (BD Biosciences) and left to adhere for 30 min at 37°C and 5% CO_2_. Subsequently, monocytes were treated as described in the Monocyte treatment section. For cell viability measurement, Calcein AM reagent (1 μg/mL; Cayman Chemical, Michigan, US) was added to the cells and incubated at 37°C and 5% CO_2_ for 30 min. Fluorescence intensity was measured by Twinkle LB 970 Microplate Fluorometer (490 nm excitation/520 nm emission; software MikroWin 2000).

### LDH Assay

100,000 cells per well were plated in a flat bottom 96-well plate (Sarstedt) and let to adhere at 37°C and 5% CO_2_ for 30 min. Subsequently, media was replaced with phenol-free RPMI 1640 medium, supplemented with 10% heat-inactivated fetal calf serum, 100 IU/mL penicillin, 10 μg/mL streptomycin and 20 mM HEPES buffer and monocytes were treated as described in Monocyte treatment section.

For cell cytotoxicity detection, 100 μL of monocyte supernatant was transferred to a fresh 96-well plate and incubated with LDH reaction mixture (Cytotoxicity Detection Kit, Roche, Mannheim, Germany) according to the manufacturer's instructions in dark at RT for 30 min. Absorbance was measured by Infinite M200 microplate reader (490 nm absorbance, 600 nm reference wavelength; software Magellan).

### Statistical Analysis

GraphPad Prism 6 (GraphPad Software Inc., San Diego, CA) was used to perform the statistical analyses. Normality of all data was analyzed by the D'Agostino-Pearson normality test. Differences between the groups were determined by non-parametric Kruskal-Wallis test which does not assume a normal distribution of the residuals followed by Dunn's *post hoc* test for the correction of multiple comparisons. A *p*-value below 0.05 was considered significant. Data are given as box-whisker plot and min to max.

## Results

### Pro-inflammatory Mediators and Lung Damage Significantly Increase in the Early Phase of Sepsis

TxT in mice increased TNF-α and MCP-1 levels in plasma, whereby the TxT+CLP group showed a significant increase compared to the control group (*p* < 0.05, data not shown). RAGE protein levels were significantly higher in TxT+CLP compared to control, both in BALF and lungs (*p* < 0.05, data not shown). Following TxT and TxT+CLP, the total protein content in bronchoalveolar lavage which is associated with the extent of lung damage significantly increased compared to control (*p* < 0.05, data not shown).

### The Ratio of Inflammatory and Patrolling Monocytes Increases in Blood, Lungs, and BALF in the Early Phase of Sepsis

For detailed examination of monocyte and macrophage subset distributions, the constituent phenotypes were characterized by their specific surface protein markers. In blood, TxT alone slightly increased levels of activated Ly6C^+^CD11b^+^Ly6G^−^CD45^+^ monocytes, whereas TxT+CLP resulted in a significant elevation compared to control (*p* < 0.05, data not shown). Regarding the subset distribution, inflammatory Ly6C^hi^CD11b^+^Ly6G^−^CD45^+^ monocyte subset expanded significantly in TxT+CLP animals (*p* < 0.05, data not shown), and, in parallel, patrolling Ly6C^lo^CD11b^+^Ly6G^−^CD45^+^ monocytes showed an equivalent decrease vs. control (*p* < 0.05, data not shown). Thus, a significant increase in the ratio between inflammatory and patrolling monocytes was observed in early phase of sepsis (*p* < 0.05, data not shown).

Similarly, activated Ly6C^+^CD11b^+^Ly6G^−^CD45^+^ monocytes were markedly more abundant in lungs following TxT+CLP compared to control lungs (*p* < 0.05, data not shown). Whereas a significant increase of the inflammatory Ly6C^hi^CD11b^+^Ly6G^−^CD45^+^ phenotype was observed in TxT+CLP group compared to control group (*p* < 0.05, data not shown), the number of patrolling Ly6C^lo^CD11b^+^Ly6G^−^CD45^+^ monocytes was equally reduced (*p* < 0.05, data not shown). Therefore, comparable to systemic monocytes, the ratio of inflammatory to patrolling monocytes increased markedly in TxT+CLP vs. control (*p* < 0.05, data not shown).

Furthermore, significantly higher emigration rates of activated Ly6C^+^CD11b^+^Ly6G^−^CD45^+^ monocytes to BALF were found in TxT and TxT+CLP compared to control (*p* < 0.05, data not shown). Regarding the inflammatory phenotypes in BALF, the inflammatory Ly6C^hi^CD11b^+^Ly6G^−^CD45^+^ monocyte subset increased in TxT and TxT+CLP compared to control (*p* < 0.05, data not shown), whereas the patrolling Ly6C^lo^CD11b^+^Ly6G^−^CD45^+^ monocyte population was reduced (*p* < 0.05, data not shown). Concurrent with this data, an increased ratio between pro-inflammatory and patrolling monocytes was found in both, TxT and TxT+CLP groups, vs. control group (*p* < 0.05, data not shown).

### The Number of Pro-inflammatory Macrophages Increases in Lungs and BALF

No significant systemic changes in Ly6C^+^F4/80^+^CD45^+^ cell counts were found after TxT and TxT+CLP compared to control (data not shown). The pro-inflammatory Ly6C^hi^F4/80^+^CD45^+^ phenotype slightly increased in TxT with further expansion in TxT+CLP vs. control but both without significance (data not shown). A decline of patrolling Ly6C^lo^F4/80^+^CD45^+^ phenotype was observed in TxT and TxT+CLP vs. control (data not shown).

Whereas, no differences in total macrophage counts were shown in the lungs of TxT and TxT+CLP, a significant increase of pro-inflammatory Ly6C^hi^F4/80^+^CD45^+^ macrophages compared to control was detected (*p* < 0.05, data not shown). The cell number of tissue repairing Ly6C^lo^F4/80^+^CD45^+^ macrophages in lungs did not change after TxT and TxT+CLP vs. control (data not shown).

Following TxT, mice displayed a slight increase of Ly6C^+^F4/80^+^CD45^+^ macrophage counts in BALF, whereas TxT with subsequent CLP did not markedly affect the cell numbers compared to control (data not shown). The inflammatory Ly6C^hi^F4/80^+^CD45^+^ phenotype expanded significantly in both, TxT and TxT+CLP group in comparison to control group (*p* < 0.05), while a significant decline of tissue repairing Ly6C^lo^F4/80^+^CD45^+^ macrophages was observed in BALF of TxT and TxT+CLP compared to control (*p* < 0.05, data not shown).

### CC16 Neutralization Is Associated With an Increase of Inflammatory Markers and Lung Damage

**Figure 1:**
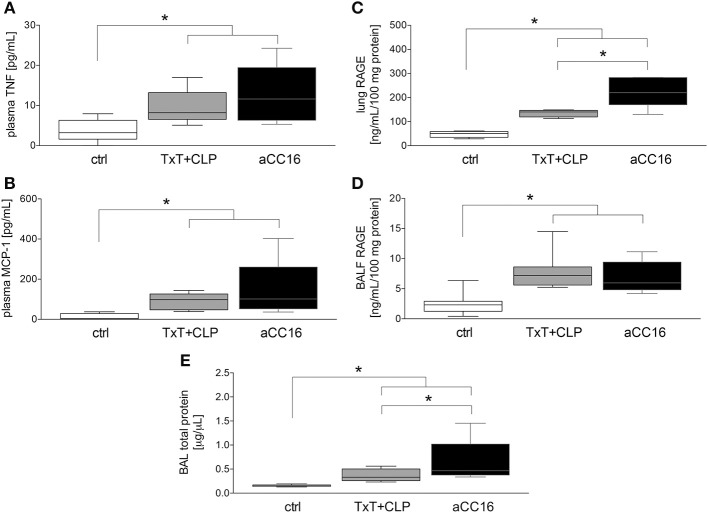
Impact of CC16 neutralization on expression levels of pro-inflammatory mediators **(A–D)** and the total pulmonary protein amount **(E)** following thoracic trauma (TxT) with cecal ligation and puncture (CLP). Plasma levels of TNF **(A)** and MCP-1 **(B)**, and RAGE protein levels in lungs **(C)** and BALF **(D)** were measured. Total protein amount was determined in BALF. Data are represented as box-whisker plot and min to max, *p < 0.05 vs. control.

To investigate the impact of CC16 on inflammatory changes and lung injury, CC16 was neutralized (CC16 Ab) in mice undergoing TxT and subsequent CLP. TxT+CLP induced a significant systemic increase of pro-inflammatory TNF-α and MCP-1 levels compared to control (*p* < 0.05) with a trend to a further increase in animals that underwent CC16 neutralization (Figures 1A,B). Whereas, protein concentrations of RAGE in both lungs (C) and BALF (D) were significantly increased after TxT+CLP vs. control, CC16 neutralization significantly increased RAGE in the lungs after TxT+CLP (*p* < 0.05, Figures 1C,D). With regards to lung tissue damage, total protein content, that was measured in the BALF, and was significantly increased after TxT+CLP vs. control, with a further significant increase in the TxT+CLP group after CC16 neutralization vs. reference TxT+CLP group (*p* < 0.05, Figure 1E).

### CC16 Modulates Phenotypic Distribution of Monocytes and Macrophages

**Figure 2:**
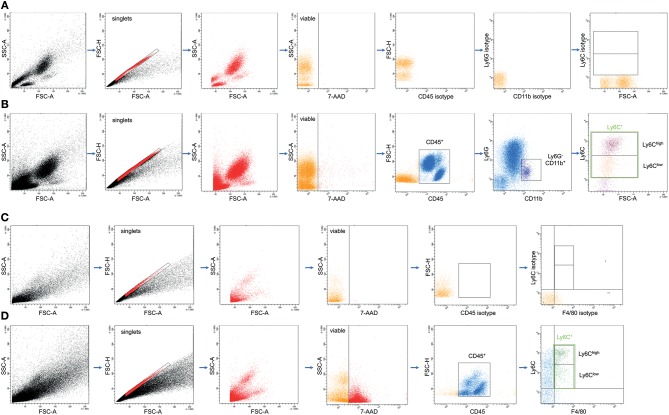
Representative gating strategy for the flow cytometric analyses and evaluation of different monocyte subsets in whole blood **(A,B)** and macrophage subsets in lungs **(C,D)** as dot plot analyses is shown. **(A,C)** are showing the gating upon staining with isotype control antibodies, while **(B,D)** show results from the staining with specific antibodies as described in the material and method section.

**Figure 3:**
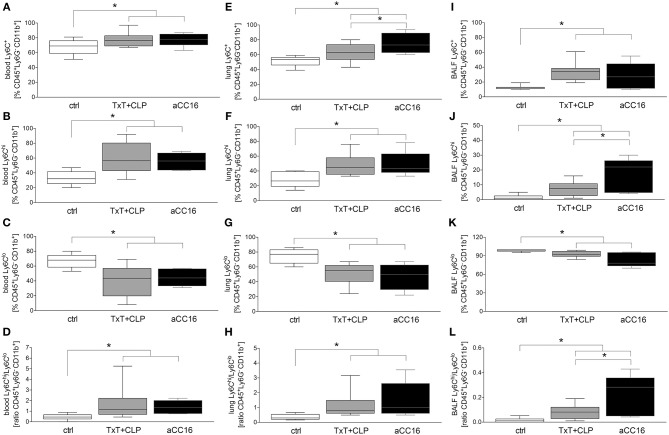
Impact of CC16 neutralization on distribution of monocyte subsets following thoracic trauma (TxT) with cecal ligation and puncture (CLP). Activated monocyte count (Ly6C^+^) as well the pro-inflammatory (Ly6C^hi^) and patrolling (Ly6C^lo^) subsets and the ratio between them were assessed in blood **(A–D)**, lungs **(E–H)** and BALF **(I–L)** by flow cytometry. Data are represented as box-whisker plot and min to max, *p < 0.05 vs. control.

The effect of CC16 on the subset distribution of monocytes and macrophages was analyzed. The representative gating for the data analyses is shown in Figure 2. Total counts of activated Ly6C^+^CD11b^+^Ly6G^−^CD45^+^ monocytes in blood increased significantly after TxT+CLP compared to control, while CC16 neutralization did not show any significant impact on this increase compared with the TxT+CLP group (*p* < 0.05, Figure 3A). Similarly, although inflammatory Ly6C^hi^CD11b^+^Ly6G^−^CD45^+^ monocytes became significantly more abundant, and the patrolling Ly6C^lo^CD11b^+^Ly6G^−^CD45^+^ monocytes displayed a significant decline in TxT+CLP animals vs. control, CC16 neutralization did not affect this subset distribution (*p* < 0.05, Figures 3B,C). The ratio of pro-inflammatory to patrolling monocytes was significantly increased in both TxT+CLP groups vs. control (*p* < 0.05, Figure 3D).

TxT+CLP induced a significantly increased migration of Ly6C^+^CD11b^+^Ly6G^−^CD45^+^ monocytes to the lungs (*p* < 0.05, Figure 3E). CC16 neutralization markedly reinforced this effect, and significantly enhanced the presence of Ly6C^+^CD11b^+^Ly6G^−^CD45^+^ monocytes in the lungs after TxT+CLP compared to the TxT+CLP reference group (*p* < 0.05, Figure 3E). However, compared to control, the significant increase of pro-inflammatory Ly6C^hi^CD11b^+^Ly6G^−^CD45^+^ and a respective decrease of patrolling Ly6C^lo^CD11b^+^Ly6G^−^CD45^+^ monocytes were not significantly modulated by CC16 neutralization after TxT+CLP (*p* < 0.05, Figures 3F,G). Thus, the ratio between Ly6C^hi^CD11b^+^Ly6G^−^CD45^+^ to Ly6C^lo^CD11b^+^Ly6G^−^CD45^+^ monocytes was significantly increased in both TxT+CLP and the TxT+CLP group undergoing CC16 neutralization compared to the control (Figure 3H).

In BALF, a significant expansion of activated Ly6C^+^CD11b^+^Ly6G^−^CD45^+^ monocytes after TxT+CLP was detected (*p* < 0.05, Figure 3I). CC16 neutralization after TxT+CLP did not change this increase compared to control (*p* < 0.05, Figure 3I). The pro-inflammatory Ly6C^hi^CD11b^+^Ly6G^−^CD45^+^ phenotype was significantly more abundant in TxT+CLP vs. control (*p* < 0.05, Figure 3J), while CC16 neutralization further enhanced the migration of inflammatory monocytes into the BALF showing a significant increase compared with the TxT+CLP group (*p* < 0.05, Figure 3J). The counts of patrolling Ly6C^lo^CD11b^+^Ly6G^−^CD45^+^ monocytes significantly declined in TxT+CLP vs. control, while CC16 neutralization did not significantly further impact this monocyte subset decrease after TxT+CLP (*p* < 0.05, Figure 3K). TxT+CLP induced a significant increase on the ratio of pro-inflammatory to patrolling monocytes compared to control (*p* < 0.05, Figure 3L), while a further significant increase after CC16 neutralization vs. TxT+CLP reference group was detected (*p* < 0.05, Figure 3L).

**Figure 4:**
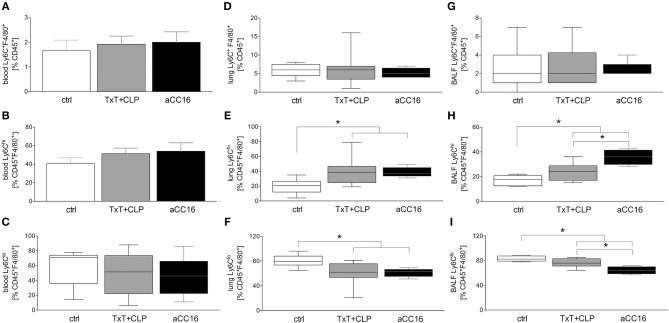
Impact of CC16 neutralization on distribution of macrophage subsets following thoracic trauma (TxT) with cecal ligation and puncture (CLP). Amount of macrophages (Ly6C^+^F4/80^+^) as well as of the pro-inflammatory (Ly6C^hi^) and patrolling (Ly6C^lo^) subsets was assessed in blood **(A–C)**, lungs **(D–F)** and BALF **(G–I)** by flow cytometry. Data are represented as box-whisker plot and min to max, *p < 0.05 vs. control.

Systemic Ly6C^+^F4/80^+^CD45^+^ cells were not markedly changed after TxT+CLP or intervention with aCC16 (Figure 4A). Neither the pro-inflammatory Ly6C^hi^F4/80^+^CD45^+^ phenotype (Figure 4B) nor the patrolling Ly6C^lo^F4/80^+^CD45^+^ phenotype (Figure 4C) were changed.

With regards to local influence of CC16 after TxT+CLP, in lungs, Ly6C^+^F4/80^+^CD45^+^ macrophage levels remained stable in TxT+CLP compared to control (Figure 4D). However, the inflammatory macrophage subset expanded significantly after TxT+CLP vs. control (*p* < 0.05, Figure 4E), whereas the anti-inflammatory phenotype decreased significantly after TxT+CLP (*p* < 0.05, Figure 4F). CC16 neutralization did not have a significant impact on macrophage subset redistribution after TxT+CLP in lungs (Figures 4D–F).

Total macrophage counts in BALF did not change after TxT+CLP, neither did CC16 neutralization change their levels (Figure 4G). However, pro-inflammatory Ly6C^hi^F4/80^+^CD45^+^ macrophages elevated significantly, while patrolling Ly6C^lo^F4/80^+^CD45^+^ macrophages declined significantly in TxT+CLP or TxT+CLP with CC16 neutralization compared to control (*p* < 0.05, Figures 4H,I). CC16 neutralization significantly increased the percentage of pro-inflammatory macrophages and reduced significantly the percentage of patrolling macrophages after TxT+CLP compared to the TxT+CLP reference group (*p* < 0.05, Figures 4H,I).

### CC16 Inhibits Migratory Capacity and TGF-β1 Expression in CD14*^+^* Monocytes *ex vivo*

**Figure 5:**
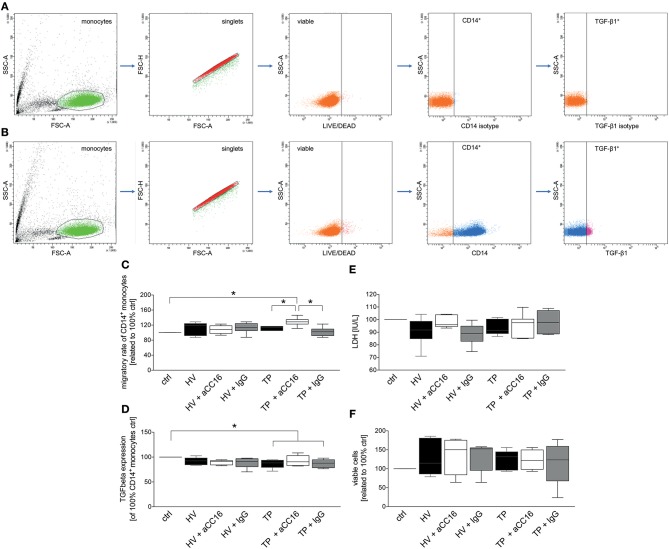
Representative gating strategy for the flow cytometric analysis and evaluation of isolated CD14^+^ monocytes with regard to their TGF-β1 expression as dot plot analysis is shown. **(A)** is showing representative isotype staining controls, while **(B)** shows representative gating after staining with specific antibodies. **(C–F)** show effects of serum samples obtained from healthy volunteers (HV) or trauma patients (TP) and CC16 on CD14^+^ monocytes behavior. CD14^+^ monocytes isolated from HV were incubated with sera obtained from HV or TP. Cell culture medium was applied alone (control) or with/without prior intervention/neutralization with either CC16 antibody (aCC16) or IgG control antibody (IgG) in sera from HV or TP. Migratory capacity toward MCP-1 **(C)**, TGF-β1 expression **(D)**, and the impact of sera on monocyte cytotoxicity **(E)** and viability **(F)** were evaluated. Data are represented as box-whisker plot and min to max, *p < 0.05 vs. indicated.

To examine the impact of CC16 on monocytes under septic conditions, systemic monocytes were isolated from healthy volunteers and subsequently stimulated with sera from HV or TP (with as well as without septic complications), since the last are known to contain higher levels of CC16 compared to control or trauma patients without complications (42). We have determined CC16 concentrations in samples of trauma patients and healthy volunteers. We found that CC16 was significantly increased in sera obtained from traumatized patients compared to those obtained from healthy volunteers (28.09 ± 4.60 vs. 15.18 ± 1.25 ng/mL, *p* < 0.05; data not shown). Regarding the migratory rate, stimulation of CD14^+^ monocytes with sera from HV did not show any changes compared to control and the neutralization of CC16 or application of isotype antibody in these sera did not affect the migration either (Figure 5C). Although the migration of CD14^+^ monocytes treated with sera from TP remained unchanged vs. control and HV, CC16 neutralization resulted in significantly higher migration rates toward MCP-1 compared to control, stimulation with sera alone or with IgG (*p* < 0.05, Figure 5C). Intracellular TGF-β1 expression showed no significant changes following treatment with sera from HV vs. control (Figure 5D). Stimulation of monocytes with sera from TP significantly declined TGF-β1 expression, and administration of CC16 neutralizing antibodies recovered TGF-β1 level to the baseline (*p* < 0.05, Figure 5D). Furthermore, TNF-α and IL-6 levels in the supernatants obtained from human monocytes that were treated with sera were determined. Stimulation of CD14^+^ monocytes with sera obtained from HV and TP did not induce any significant impact on TNF-α nor IL-6 levels (data not shown). TNF-α concentration of the control was 30.06 ± 4.60 pg/mL. Following treatment with sera from HV, TNF-α level was comparable at 32.42 ± 6.90 pg/mL, whereas CC16 neutralization in those sera did not lead to a significant decrease (24.70 ± 5.306 pg/mL). Supernatants from cells that were stimulated with TP sera have shown comparable concentrations of TNF-α to those obtained after incubation with TP sera upon CC16 neutralization (22.01 ± 3.20 vs. 30.18 ± 3.66 pg/mL). Control IL-6 concentration was 46.96 ± 12.31 pg/mL. Treatment with sera from HV did not change the IL-6 level, which was 50.13 ± 19.57 pg/mL and which also stayed stable after CC16 neutralization (40.48 ± 13.14 pg/mL). Comparable results were found in supernatants from TP samples (40.97 ± 6.436 pg/mL) and the corresponding CC16 neutralized sample also (45.84 ± 6.66 pg/mL). Further, we examined the cytotoxic potential of CC16 analyzing the release of LDH. Here, the stimulation with neither sera from HV nor TP changed the LDH release compared to untreated control (Figure 5E). Finally, treatment with both sera and sera with neutralized CC16 or IgG did not show significant changes in the viability of CD14^+^ monocytes (Figure 5F).

## Discussion

Since blunt chest injury with ongoing excessive pro-inflammatory immune response entails high risk for the development of secondary complications with limited therapeutic options, the unveiling of underlying mechanisms is necessary (43, 44). Here, we discuss the dynamic changes in monocyte and macrophage subsets and uncover the potentially protective role of the anti-inflammatory endogenous CC16 in the very early phase of sepsis development following thoracic injury. We confirmed the anti-inflammatory potential of CC16 in the early phase of sepsis-induced ALI following blunt chest injury. Its local neutralization after thoracic trauma increased the immigration of pro-inflammatory cell phenotypes to the lungs, and was accompanied by increased total protein levels in BALF, indicating the loss of epithelial lung integrity, and thus lung damage. Concurrently, systemic elevation of humoral pro-inflammatory mediators was observed. This is in line with our recent study, where early increased lung infiltration with neutrophils and lung injury in this model was shown (45). However, in that study, 24 h post-CLP, the lung injury was ameliorated and the lungs have exhibited no further increase in neutrophilic infiltration after CC16 neutralization (45). Thus, CC16 may first reduce a necessary early pro-inflammatory immune response for tissue repair, and at a later time point, may contribute to the amelioration of the lung injury. Although the mechanism is still not clear, the observed lung injury could be caused by the paralleled enhanced lung infiltration with neutrophils. This assumption is supported by Lerman et al. where neutrophil extravasation and tissue infiltration in murine CLP-induced sepsis were inhibited by blocking or deletion of α3β1 and paralleled by significantly reduced mortality (46).

Following infectious or non-infectious stimuli, alveolar macrophages are, among other cells, the first to be involved in the early immune response, initiating the inflammatory cascade and secretion of pro-inflammatory mediators (20–23). We have shown that thoracic injury followed by CLP increased systemic levels of TNF-α; which is an important indicator of sepsis development (47). Neutralization of endogenous CC16 forced further increase of TNF-α, indicating anti-inflammatory capacity of CC16 in the present model, and confirming in general its anti-inflammatory character. In turn, elevated TNF-α level is also one of the factors inducing MCP-1 expression by a variety of cell types (48). Consistent with literature, we have shown that TNF-α increase after thoracic trauma and CLP was paralleled by a systemic MCP-1 increase. CC16 neutralization led similarly to TNF-α to a further systemic increase of MCP-1. Whether this further MPC-1 increase is caused directly by CC16 or indirectly by an increase of TNF-α or other factors still remains to be elucidated in future studies.

Further, we have shown that expression of RAGE significantly increased in lungs and BALF of septic mice, whereas CC16 neutralization led to further significant increase in the lungs. In a clinical study, both CC16 and RAGE were identified as plausible biomarkers for ARDS in patients with severe sepsis (49), but whether RAGE positively or negatively regulates the immune response seems to differ according to the inflammatory mechanism (50, 51). Moreover, we have shown that blunt chest injury itself increased the protein levels in BALF and subsequent CLP did not cause a further increase after 6 h. This does not mean that sepsis did not have an impact on lung epithelial integrity, but lungs are at this time point affected by thorax trauma directly and abdominal-induced sepsis may take longer to affect the lung epithelial integrity than could be seen in the observation period. Upon CC16 neutralization, total protein level in BALF further increased, suggesting a positive impact of CC16 in lung epithelial injury.

Following sepsis-induced ALI after blunt chest trauma, a significant increase of monocyte counts was observed in blood and lungs, as well as in the BALF. The characterization of monocytes has uncovered significantly more pro-inflammatory monocytes compared to a marked decline of the patrolling phenotype. Since MCP-1 is the pivotal regulator of monocyte recruitment to the site of injury (12), the observed elevated MCP-1 levels in septic mice may be the key factor for the excessive infiltration of lungs with pro-inflammatory monocytes, whereby the disrupted lung epithelial integrity may contribute to the higher monocyte content in BALF as well. Upon CC16 neutralization, increased monocyte emigration to the lungs was observed, indicating the anti-migratory potential of CC16. To substantiate our assumption, we isolated monocytes from healthy volunteers and stimulated them with sera obtained from healthy volunteers or from trauma patients, since we have shown in previous studies that increased systemic concentrations of CC16 correlate with the development of secondary respiratory complications following traumatic injury in patients and is lowered in healthy individuals (31, 42, 52). CC16 neutralization in patient's sera before monocyte stimulation led to a significant increase of monocyte chemotaxis toward MCP-1 but the mechanism still remains to be elucidated. However, although CC16 was neutralized, monocytes from TP+aCC16 display elevated migration compared to controls. Serum from trauma patients contains other mediators beside CC16, which may change the migratory behavior of monocytes, thus further mediators such as IL-6 or RANTES that potentially increase the migratory capacity of monocytes are probably concurrently present in the blood from trauma patients (53–56). In a further *ex vivo* experiment, we have shown unaffected viability of isolated monocytes and their release of lactate dehydrogenase following treatment with sera, suggesting no cytotoxic effects of CC16. Interestingly, stimulation of isolated human monocytes with sera obtained from trauma patients led to a significant decrease of TGF-β1 expression, whereas neutralization of CC16 recovered the levels to the baseline. Although this was unexpected, it was already reported in rodent models of lung fibrosis that CC16 contributes to diminished TGF-β1 levels, however, the mechanism still remains elusive (37, 57). Here, some studies indicate that CC16 suppresses TGF-β1 expression via MPP-9 inhibition (34, 58).

Regarding macrophage distribution, we observed elevated levels of pro-inflammatory macrophages paralleled by decline of anti-inflammatory phenotype in lungs and BALF. CC16 neutralization reinforced the observed changes, whereas lung infiltration remained unaffected. Since resident alveolar macrophages have been described to have anti-inflammatory properties in steady state and, upon infection or injury, they display a phenotypic shift and gain pro-inflammatory features (18), we hypothesized that CC16 neutralization may contribute to macrophage polarization toward the pro-inflammatory phenotype. Whether CC16 in fact suppresses an exaggerated transition to pro-inflammatory macrophages remains to be further elucidated by future studies.

## Limitations

Showing an isolated CLP group with performed interventions would further increase the relevance of our results. However, since the scope of the present study was to elaborate the role of CC16 in the underlying double-hit model, this approach was not considered. In humans, it is well-known that secondary stimuli (e.g., surgeries and infections) following chest trauma contribute to the development of secondary pulmonary complications, including ALI and ARDS (44). In mice, it was already shown that pulmonary contusion primes the systemic innate immune response to the LPS challenge, increasing inflammation and worsening lung injury compared to injury or LPS application alone (59). Moreover, recently we have shown that isolated blunt chest trauma in mice was not enough to mimic human conditions since the ongoing pro-inflammatory response decreased to baseline within 24 h, and that combining blunt chest trauma with CLP led to pulmonary changes that were characteristic for ALI (41). However, the mechanism is still unknown, and it remains to be elucidated whether either direct local tissue injury and the subsequent pro-inflammatory response, or the second hit with excessive pro-inflammatory response and remote organ damage contribute more to the ALI development (41, 60). However, literature indicates that the combination of both hits contributes to the increased pro-inflammatory response following double-hit trauma (61–64). Thus, the question of whether CC16 neutralization would affect the isolated CLP animals in the same way as in the TxT+CLP group remains unanswered. Furthermore, showing CC16 levels in all experimental groups at the timing of therapy would support our findings. Yet, due to ethical reasons with regard to animal protection, such analyses were not possible. Following the principle of 3Rs (Replacement, Reduction, Refinement) the number of animals per each group was limited to 8. Following severe thoracic injury, human trauma patients mostly require mechanical ventilation, whereas mice were spontaneously breathing in our experimental settings. Thus, the impact of mechanical ventilation following chest injury could not be considered. Moreover, although mouse models are key tools for studying different pathophysiologies, the immune response between mouse and human differs and the applied treatment cannot be directly translated into human settings. We could not show the impact of CLP on lung integrity, and we consider the short observation period as a further limitation that could lead to negative results. Similarly, monocytes and recruited and resident alveolar macrophages seem to have specific functions in a time-dependent manner. Thus, the right time frame for the examination of monocyte and macrophage function is essential and has to be examined further. In flow cytometric analysis, the chosen markers did not distinguish between recruited and resident alveolar macrophages and this has to be examined in future studies also. Furthermore, although CC16 neutralization increased pro-inflammatory monocyte and macrophages phenotypes, whether CC16 directly contributes to exaggerated transition to pro-inflammatory macrophages still remains to be elucidated. A longer observation period of up to 7 days would bring clarity to the beneficial or negative effects of CC16. Additionally, the distribution of neutrophil and monocyte/macrophage subsets should be evaluated as well. Comparing this with the extent of the lung injury would clarify whether CC16 has either a negative or positive effect on outcomes. It would be interesting to know whether in case of positive effects, CC16 would improve only the lung injury and pro-inflammatory immune response or whether the survival would be improved as well. To confirm the above-discussed potential results, recombinant CC16 therapy should be applied as well. In *in vitro* studies, we pooled sera from only ten trauma patients without secondary complications and 10 trauma patients who developed sepsis in a later course, and thus, a larger sample size may clarify the results. Although CC16 is known to have anti-inflammatory properties, we have shown recovered TGF-β1 protein expression levels in CD14^+^ monocytes following treatment with a trauma patient's sera. It is possible that TGF-β1 expression is inhibited indirectly by another mechanism and this has to be evaluated in further studies as well.

## Data Availability Statement

All relevant datasets for this study are contained in the manuscript.

## Ethics Statement

This studies involving human participants were reviewed and approved by Institutional Ethical Committee of the University Hospital Frankfurt, Goethe-University, Germany. The patients/participants provided their written informed consent to participate in this study. The animal study was reviewed and approved by Veterinary Department of the Regional Council in Darmstadt, Germany (Regierungspräsidium Darmstadt, Hessen.

## Author Contributions

BR: conceptualization, supervision, and project administration. AJ, PS, NB, BX, and BR: methodology. AJ: validation, data curation, and writing—original draft preparation. BR and AJ: formal analysis. AJ, PS, NB, and BX: investigation. BR, SW, FH, and IM: resources. AJ, JV, SE, and BR: writing—review and editing. AJ and BR: visualization. BR, FH, and SW: funding acquisition.

### Conflict of Interest

The authors declare that the research was conducted in the absence of any commercial or financial relationships that could be construed as a potential conflict of interest. The reviewer CE declared a shared affiliation, with no collaboration, with one of the authors, FH, to the handling editor.

## Abbreviations

Ab: antibody
ALI: acute lung injury
ARDS: acute respiratory distress syndrome
BALF: bronchoalveolar lavage fluid
CC: club cell protein
CLP: cecal ligation and puncture
HV: healthy volunteers
ISS: injury severity score
LPS: lipopolysaccharide
Ly: lymphocyte antigen
MCP: monocyte chemoattractant protein
MPP: matrix metalloproteinase
RAGE: receptor for advanced glycation endproducts
TNF: tumor necrosis factor
TP: trauma patient
TxT: thoracic trauma.
